# The thyroid gland and the process of aging; what is new?

**DOI:** 10.1186/1756-6614-5-16

**Published:** 2012-11-24

**Authors:** Adam Gesing, Andrzej Lewiński, Małgorzata Karbownik-Lewińska

**Affiliations:** 1 Department of Oncological Endocrinology, Medical University of Lodz, Zeligowski St., No. 7/9, Lodz, 90-752, Poland; 2 Department of Endocrinology and Metabolic Diseases, Polish Mother's Memorial Hospital – Research Institute, Rzgowska St., No. 281/289, Lodz 93-338, Poland; 3 Department of Endocrinology and Metabolic Diseases, Medical University of Lodz, Rzgowska St., No. 281/289, Lodz, 93-338, Poland

**Keywords:** Thyroid gland, Aging, Subclinical thyroid dysfunction, Thyroid cancer, Longevity

## Abstract

The endocrine system and particular endocrine organs, including the thyroid, undergo important functional changes during aging. The prevalence of thyroid disorders increases with age and numerous morphological and physiological changes of the thyroid gland during the process of aging are well-known. It is to be stressed that the clinical course of thyroid diseases in the elderly differs essentially from that observed in younger individuals, because symptoms are more subtle and are often attributed to normal aging. Subclinical hypo- and hyperthyroidism, as well as thyroid neoplasms, require special attention in elderly subjects. Intriguingly, decreased thyroid function, as well as thyrotropin (TSH) levels – progressively shifting to higher values with age – may contribute to the increased lifespan.

This short review focuses on recent findings concerning the alterations in thyroid function during aging, including these which may potentially lead to extended longevity, both in humans and animals.

## Introduction

The endocrine system and particular endocrine organs, including the thyroid gland, undergo – similarly to other organ systems – crucial functional changes with aging. Numerous morphological and physiological changes of the thyroid during the process of aging are well-known [1-3]. A specificity of thyroid diseases in the elderly, differing essentially from that observed in younger subjects, relies on the presence of more subtle symptoms which are often attributed to normal aging. Therefore, subclinical hypo- and hyperthyroidism, as well as thyroid neoplasms, the prevalence of which increases with age, require special attention in elderly subjects. Interestingly, altered thyroid function may contribute to the extended longevity. The present review focuses on the newest findings concerning the alterations in thyroid function during the process of aging.

### Thyroid dysfunction with aging

The process of aging affects both the prevalence and clinical presentation of hypo- and hyperthyroidism. Importantly, subclinical disturbances of thyroid function are more frequent than overt diseases in general population, as well as in elderly people [4,5]. Consistently, the prevalence of subclinical hypothyroidism, which is characterized by normal free thyroxine (FT_4_) and elevated thyrotropin (TSH) levels, increases with aging [6-12] and ranges from 3 to 16% in individuals aged 60 years and older [13].

Although it is known that overt thyroid disorders negatively affect physical and cognitive function in elderly people – for example, overt hypothyroidism is associated with the impairment of attention, concentration, memory, perceptual functions, language, and executive functions [14], subclinical hypothyroidism is not associated with impairment of physical and cognitive function or depression in individuals aged 65 years and older, as compared to euthyroidism [15]. Also Park et al. [16] have demonstrated that subclinical hypothyroidism in elderly subjects is neither associated with cognitive impairment, depression, poor quality of life nor with metabolic disturbances. On the other hand, other studies demonstrated the presence of – at least – mild cognitive impairment in people with subclinical hypothyroidism at mean age under 65 years (reviewed in [17]). Furthermore, as reported by de Jongh et al. [15], subclinical hypothyroidism was also not associated with the increased overall mortality risk. Similar findings were shown by Rodondi et al. [18] who analyzed data from numerous large prospective cohorts and demonstrated that total mortality was not increased in subjects with subclinical hypothyroidism, although the risk of coronary heart disease (CHD) events and of CHD mortality increased with TSH levels 10 mIU/l or higher. Nevertheless, it should be emphasized that this analysis regarded numerous different populations (cohorts) which consisted of not only elderly people and that the effect in question, i.e. of increasing TSH level on CHD incidents was not influenced by age [18].

Undoubtedly, there are obvious indications for treatment of overt hypothyroidism. On the other hand, indications for treatment of subclinical hypothyroidism are still controversial. Despite improvement of lipid profile due to treatment of subclinical hypothyroidism, there is no clear evidence that this beneficial effect can be associated with decreased cardiovascular or all-cause mortality in elderly patients [19]. Furthermore, Parle et al. [20] have reported that L-thyroxine replacement therapy does not improve cognitive function in elderly individuals with subclinical hypothyroidism. When the natural history of subclinical hypothyroidism was evaluated in the elderly, the final results depended on the presence or absence of thyroid antibodies and on that to what extent TSH concentration was increased. Thus, a quite high rate of reversion of subclinical hypothyroidism to euthyroid status in adults aged at least 65 years with lower baseline TSH levels and antithyroid peroxidase antibody (TPOAb) negativity was observed [21]. In turn, higher TSH level and TPOAb positivity were independently associated with lower chance of reversion to euthyroidism [21]. Moreover, TSH levels ≥ 10 mIU/l were independently associated with progression to overt hypothyroidism [21]. Similar findings, showing that higher baseline TSH levels are associated with progression from subclinical to overt hypothyroidism and that higher TSH level (> 8 mIU/l) is a predictive value for development of overt hypothyroidism, were recently reported by Imaizumi et al. [22]. On the other hand, there is strong evidence that thyroid hypofunction may contribute to increased lifespan (see further in the text). Therefore, taking into account all mentioned observations, the replacement therapy with L-thyroxine is not uniformly recommended in elderly people with subclinical hypothyroidism.

In turn, subclinical hyperthyroidism, characterized by serum TSH levels below lower limit of the reference range and normal serum FT_4_ levels, is observed in about 8% of individuals aged 65 years and older [23]. Subclinical hyperthyroidism may be associated in older adults with decreased bone mineral density and fractures [24], or cognitive impairment [23] (reviewed in [25]). Furthermore, subclinical hyperthyroidism is associated with increased risk of total, as well as CHD mortality and atrial fibrillation (AF) incidents [26]. The highest risks of CHD mortality and AF are observed in the case of TSH levels lower than 0.1 mIU/l [26]. Unexpectedly, de Jongh et al. [15] have reported that subclinical hyperthyroidism is not associated with impairment of physical and cognitive function or depression in elderly people, aged 65 years and older. These authors have also demonstrated that subclinical hyperthyroidism is not associated with the increased overall mortality risk [15]. Such results are quite difficult to explain. Presumably, that ambiguity in observations may result from differences in the number of individuals enrolled in particular studies or from follow-up duration. Interestingly, Rosario [27] has recently shown that progression of subclinical hyperthyroidism to overt hyperthyroidism in elderly patients is an uncommon observation. Nevertheless, since subclinical hyperthyroidism (and obviously, overt hyperthyroidism with increased T_4_ level) may lead to increased risk of total, as well as CHD mortality, patients older than 65 years, with low TSH levels – particularly in case of toxic multinodular goitre or a solitary autonomous thyroid nodule – require proper medical treatment (e.g. [11]).

It should also be stressed that during aging, gender-specific alterations in TSH and free thyroid hormone levels were observed [28]. Namely, with increasing age in males there were decreases in free thyroid hormones but not in TSH concentrations. In turn, in females, the free thyroid hormone levels were not changed with aging but TSH level increased in age-dependent manner [28].

Most recent results indicate that even in euthyroid older men with normal levels of TSH, differences in FT_4_ levels within the normal range predict specific health outcomes relevant to aging. For example, higher FT_4_ within the normal range was independently associated with frailty in euthyroid men aged ≥70 years [12]. Moreover, higher FT_4_ levels within the normal range were associated with lower hip bone mineral density, increasing bone loss and fracture risk in postmenopausal women [29]. Therefore, it seems that further studies are required to explain whether higher FT_4_ levels contribute causally (or not) to the above mentioned poorer health outcomes. Moreover, it is of interest to clarify whether FT_4_ levels in the low-normal range could be considered as potential biomarkers for healthy aging [12].

Although numerous studies demonstrate that the increased TSH level resulting from subclinical hypothyroidism further rises with aging [6-12], other findings suggest that aging is associated – in the absence of any thyroid disease – with lower TSH levels [30-35]. It has been known that TSH secretion in response to thyrotropin-releasing hormone (TRH) is reduced in aging individuals, and serum TSH level is usually lower in older than in young people in response to decreased thyroid hormone concentrations, suggesting a certain level of insensitivity of thyrotrophic cells in anterior pituitary, occurring with age; moreover, nocturnal surge of TSH is – to various degree – lost in the elderly (reviewed in [1]). On the other hand, Bremner et al. [10] have recently reported that the TSH increase – observed by other authors during aging – seems to be a consequence of age-related alteration in the TSH set point or reduced TSH bioactivity. Interestingly, the largest TSH increase is observed in people with the lowest TSH at baseline, and, in turn, people with higher baseline TSH levels had proportionally smaller increases in TSH concentrations [10]. It is worth adding that TRH and FT_4_ serum levels do not differ between young, middle-aged and elderly subjects [34].

### Thyroid dysfunction and longevity

As it has been mentioned above, the alterations in levels of hormones related to pituitary-thyroid axis are associated with the process of aging and, thus, may impact longevity. However, a direction of these changes, which may lead to increased lifespan, still seems to be not fully determined [6-12,30-35].

One should emphasize that the most striking findings concerning potential contribution of TSH and thyroid hormones to lifespan regulation, were obtained in the studies performed on centenarians (and almost centenarians). In 2009, Atzmon et al. [7] published the results of studies on thyroid disease-free population of Ashkenazi Jews, characterized by exceptional longevity (centenarians). They have observed higher serum TSH level in these subjects as compared to the control group consisted of younger unrelated Ashkenazi Jews, as well as to another control group obtained from The National Health and Nutrition Examination Survey (NHANES) program of studies [7]. Therefore, these findings appear to support previous observations, indicating that serum TSH shifts progressively to higher levels with age (e.g., [36]). Moreover, the authors have observed an inverse correlation between FT_4_ and TSH levels in centenarians and Ashkenazi controls, and finally, they have distinctly concluded that increased serum TSH is associated with extreme longevity [7]. In another study, a role of genetic background, potentially responsible for the above-mentioned changes, was assessed [37]. It turned out that two (2) single nucleotide polymorphisms (SNPs) in TSH receptor (TSHR) gene, namely rs10149689 and rs12050077, were associated with increased TSH level in the Ashkenazi Jewish centenarians and their offspring [37].

The above-mentioned inverse correlation between FT4 and TSH in centenarians may suggest a potential role of decreased thyroid function in lifespan regulation, leading to remarkable longevity. Such a hypothesis seems to have been confirmed by the findings obtained in the Leiden Longevity Study, demonstrating the associations between low thyroid activity and exceptional familial longevity [38].

In turn, Corsonello et al. [39] have demonstrated that age is associated with a decrease in free triiodothyronine (FT_3_) and FT_4_ but not with increased TSH levels. Moreover, children and nieces/nephews of centenarians had lower FT_3_, FT_4_ and TSH levels as compared to the age-matched subjects [39]. It may, at least partially, confirm an important role of low thyroid function in the regulation of lifespan.

It should be stressed that reduced thyroid function with low levels of T_4_ is associated with extended longevity also in animals [40-42]. For example, a very severe thyroid hypofunction with reduced core body temperature, as observed in Ames dwarf (df/df) and Snell mice (characterized by mutations at the Prop-1 and Pit-1 gene, respectively, and demonstrating a lack of growth hormone (GH), prolactin and TSH), is considered to substantially contribute to remarkable longevity in these rodents [40]. Furthermore, severe hypothyroid Ames dwarfs and mice with targeted disruption of the growth hormone receptor/growth hormone binding protein gene (GH receptor knockout; GHRKO) with mild thyroid hypofunction, have decreased thyroid follicle size which may explain decreased thyroid hormone levels in these mutants [43].

Concluding, the findings in animals are consistent with the results obtained in humans and may confirm a relevant role of thyroid hypofunction in lifespan extension.

### Thyroid cancerogenesis and aging processes

The prevalence of thyroid nodules and thyroid neoplasms is increased in the elderly. Among elderly people, males are at higher risk of cancer and thyroid cancer is more aggressive in men than in women [44].

Papillary thyroid carcinoma (PTC) is the most common endocrine malignant neoplasm in the older individuals. Women are affected by PTC two to three times more often than men [45]. Nevertheless, female-to-male ratio seems to decline with the process of aging [45]. Importantly, the mortality rate of PTC is usually higher in the elderly [46]. Presumably, it is a consequence of increased mitotic activity of these tumors and increased likelihood of distant metastases [46]. It is known that in general population patients with aggressive variants of PTC have higher risk for the metastatic disease development [47]. The potential role of NDRG2 gene expression in the development and progression of PTC is also raised [48]. It is worth recalling that mutated BRAF gene is an independent predicting factor of poor outcome in PTC and is related to advanced age [49].

Follicular thyroid carcinoma (FTC) occurs also often in older people and is the second most common and the second least aggressive thyroid cancer. This cancer is more likely to metastasize hematogenously to distant sites, resulting in a worse prognosis in comparison with PTC [44].

Medullary thyroid carcinoma (MTC), which derives from the parafollicular cells (C cells) of the thyroid gland, constitutes up to 5% of all thyroid malignancies. Its sporadic form, more frequent than is familial MTC, occurs more commonly in the older population [50].

Rapidly growing and typically very aggressive anaplastic (undifferentiated) thyroid carcinoma (ATC) is rare. However, one should strongly emphasize that its prevalence is considerably higher in older than in younger people. By the time of diagnosis, most patients have widespread local invasion and distant metastases. Age appears to be a strong predictor of poor prognosis in ATC [44].

## Conclusions

The process of aging strongly affects entire endocrine system. Consistently, thyroid gland is also impacted by aging. One should emphasize that thyroid diseases-associated symptoms in the elderly people are very similar to symptoms of the normal aging. Therefore, broadening the knowledge on alterations in thyroid function, which may be observed during aging, appears to be very important and constitutes a challenge for thyroid researchers, given that some specific thyroid dysfunctions may contribute to lifespan extension.

## Competing interests

The authors declare that they have no competing interests.

## Authors’ contributions

AG wrote the draft of manuscript. AL was involved in the revision of the text. MKL supervised preparation of the final version of manuscript. All authors read and approved the final manuscript.
